# Supplementary material for: OmnibusX: A unified platform for accessible multi-omics analysis
Source: PLoS Comput Biol. 2025 Sep 8;21(9):e1013480. doi: 10.1371/journal.pcbi.1013480 (PMC12431643; doi:10.1371/journal.pcbi.1013480)
Supplement: Attachment — Submitted filename: Response to Reviewers.docx [file pcbi.1013480.s003.docx]

TO,

The Editor, Reviewers,

PLOS Computational Biology

Date: August 14, 2025

**Subject: Response to Editor and Reviewers for Manuscript PCOMPBIOL-D-25-01016**

**OmnibusX: A unified platform for accessible multi-omics analysis**

Dear Dr. Lun Hu, Dr. Ilya Ioshikhes, and Reviewers,

Thank you for your letter and for the opportunity to revise our manuscript, PCOMPBIOL-D-25-01016, titled "OmnibusX: A unified platform for accessible multi-omics analysis". We are grateful to you and the reviewers for the insightful and constructive comments. We have carefully considered all the suggestions and have revised the manuscript accordingly. Kindly help us with your further concerns (if any) to improve our paper. Below, we provide our responses to the comments and the actions we took to address them. The responses are highlighted in green.

Thank you.

Kind regards,

Huy Nguyen

---------------------------------------------------------------------------------------------------------------------

Additional Editor Comments:

Although reviewers see the value of this work, they also had several major concerns that need to be resolved before publications. Particularly, authors should revise their manuscript by clarifying the availability of offline version, the extension of validation, and discussion of future work.

We thank the editor for their constructive feedback and the time devoted to handling our manuscript. We have revised the manuscript accordingly to address the concerns raised.

Reviewers' comments:

Reviewer #1: This is an interesting paper that introduces a useful integrative tool for multi-omics data analysis. The platform could be very helpful for researchers, especially those who don’t have a background in programming. The focus on local data processing and privacy is also a strong point, especially for clinical or sensitive data.

We thank the reviewer for their positive and encouraging feedback. We appreciate the recognition of our platform’s utility for researchers without a programming background, as well as the emphasis on local data processing and privacy. These aspects are core design principles of our tool, and we are pleased that the reviewer found them valuable.

However, there are a few inconsistencies that should be addressed:

1. The paper stresses the importance of avoiding cloud-based tools for privacy reasons, yet it mentions that OmnibusX connects to an AWS server for user authentication and downloading reference files. Doesn’t this AWS connection breach the very data security concerns the platform claims to avoid? Clarifying whether a fully offline mode is available would help.

We thank the reviewer for raising this point. OmnibusX does not transmit user-provided datasets, analysis results, or usage logs to any external server. The AWS-hosted server connection is used solely for three purposes: (1) user authentication, (2) license validation, and (3) downloading publicly available reference files such as gene annotations or marker sets. These API calls are initiated from the user’s local machine only when required and are not persistent connections. No logs of user activity are recorded, ensuring that all scientific data and usage patterns remain entirely local. We have added a clarifying sentence to the Results section (Page 16, Lines 333–335) to address this point.

2. The authors highlight transparency, but the platform includes proprietary tools like the cell type prediction engine. It’s unclear whether users have access to the code or functionality behind these tools. More explanation would be helpful.

We thank the reviewer for the opportunity to clarify this point. OmnibusX is transparent about its analytical workflow by specifying the computational packages used—whether open-source or proprietary—the stage at which they are applied, and the parameters available for user adjustment.

The proprietary cell type prediction engine is based on a curated database of 166 cell type and subtype-specific marker gene sets, compiled from 280 publications and validated across multiple datasets and tissues. The prediction algorithm builds on the AUCell method with additional steps to remove low-level background signals, calculate enrichment scores, and assign cell labels, followed by a smoothing step for unassigned cells based on nearest-neighbor consensus. While the curated marker sets and source code are proprietary to protect intellectual property and maintain competitive advantage, the methodology, algorithmic steps, and benchmarking results are published in detail in Truong et al., 2024. Users can manually adjust predictions or apply their own custom marker sets, ensuring flexibility and reproducibility in downstream analyses.

Given that OmnibusX is a self-funded commercial product, this represents the highest level of transparency possible while safeguarding intellectual property.

We have added a clarifying paragraph to the Design and Implementation section (Page 8, Lines 166–177) to address this point.

3. The format support is a bit unclear. Seurat files are listed as supported, but later Seurat v5 is said to be unsupported. A clear list of supported formats would improve clarity.

We thank the reviewer for pointing out this ambiguity. At the time of manuscript preparation, OmnibusX supported Seurat objects up to version 4; Seurat v5 was not yet compatible. Since then, OmnibusX has been updated to fully support Seurat v5 objects.

To improve clarity, we have updated the manuscript to provide a clear list of supported file formats for each sequencing technology. This list reflects the current release of OmnibusX and includes both plain text, image, and binary formats relevant to the supported workflows.

We have added a clarifying paragraph to the Results section (Page 16-17, Lines 349–360) to address this point.

4. Batch correction is described as being applied only for visualization (e.g., UMAP). It would be helpful to explain how this enhances UMAP plots by reducing batch effects and making cell populations more comparable across samples.

We thank the reviewer for this suggestion. Batch correction in OmnibusX is applied in the principal component space prior to generating low-dimensional embeddings such as UMAP or t-SNE. This step reduces variation caused by technical or experimental differences, enabling biologically similar cells from different batches to align more closely in the embedding space. The result is a clearer and more accurate visual representation in which separation of cell populations reflects biological rather than technical differences, facilitating direct comparison across samples.

We have updated the Batch effect correction subsection (Page 9, Lines 188-196) to explicitly describe this benefit.

5. The provided link to the trial version (https://omnibusx.com/apps) was not working at the time of review. This should be checked and updated.

We thank the reviewer for noting this issue. The trial version link (https://omnibusx.com/apps) is active and functional. The downtime the reviewer experienced coincided with a scheduled server upgrade, during which our AWS instance was temporarily shut down to expand capacity for new users. This upgrade was completed within a short period, and all services have since been fully restored. We have verified that the link is currently accessible and operational.

Fixing these issues would strengthen the manuscript and improve its usability.

Reviewer #2: Thank you for the opportunity to review your manuscript, “OmnibusX: A Unified Platform for Accessible Multi-Omics Analysis.” The platform you present addresses a clear and timely need in the life sciences by offering an integrated, privacy-conscious, and user-friendly environment for multi-omics analysis.

Strengths

- The wide support for diverse omics data types, including bulk and single-cell RNA-seq, scATAC-seq, spatial transcriptomics, TCR/BCR sequencing, and ADT, is impressive and highly valuable.

- The local, code-free architecture strikes a thoughtful balance between usability and data privacy, lowering barriers for non-programmers while supporting institutional compliance.

- Community-driven features and open access to curated resources enrich the user experience and foster collaborative development.

- The team’s commitment to continuous improvement and user feedback is commendable.

We sincerely thank the reviewer for their thoughtful and encouraging assessment of our work. We appreciate the recognition of the platform’s broad support for diverse omics data types, its local, code-free architecture, and its emphasis on both usability and data privacy. We are also grateful for the acknowledgment of our community-driven features, open access curated resources, and our commitment to continuous improvement based on user feedback. These elements are central to our design philosophy, and we are pleased that the reviewer found them valuable.

Suggestions for Improvement

- Benchmarking: Including performance benchmarks and comparisons with widely used tools (e.g., Seurat, Scanpy, Galaxy) within the manuscript would help contextualize OmnibusX’s strengths.

We thank the reviewer for this suggestion. OmnibusX integrates widely used open-source packages such as Seurat and Scanpy directly within its analytical pipelines, ensuring that performance for core analyses matches that of these frameworks. For proprietary modules, including the cell type prediction engine, we have already published detailed benchmarking against state-of-the-art tools (Truong et al., 2024), demonstrating improved performance in cell annotation tasks.

The primary aim of this manuscript is to present OmnibusX as a unified, privacy-centric platform that combines these validated analytical methods with proprietary modules, interactive visualization, and workflow integration. Since the system builds upon and extends these established tools rather than replacing them, we have not included performance benchmarks for their standard methods in this work.

- Transparency: More information on proprietary modules (especially the cell-type annotation engine) would improve clarity.

References to validation studies or technical documentation would be helpful.

We thank the reviewer for the opportunity to clarify this point. OmnibusX is transparent about its analytical workflow by specifying the computational packages used—whether open-source or proprietary—the stage at which they are applied, and the parameters available for user adjustment. Detailed technical documentation, including the full list of integrated open-source packages (e.g., Scanpy, Seurat), their role within the preprocessing and downstream analysis pipelines, and the interaction between proprietary and open-source components, is publicly available at <https://omnibusx.com/resources#user-guide>.

The proprietary cell type prediction engine is based on a curated database of 166 cell type and subtype-specific marker gene sets, compiled from 280 publications and validated across multiple datasets and tissues. The prediction algorithm builds on the AUCell method with additional steps to remove low-level background signals, calculate enrichment scores, and assign cell labels, followed by a smoothing step for unassigned cells based on nearest-neighbor consensus. While the curated marker sets and source code are proprietary to protect intellectual property and maintain competitive advantage, the methodology, algorithmic steps, and benchmarking results are published in detail in Truong et al., 2024. Users can manually adjust predictions or apply their own custom marker sets, ensuring flexibility and reproducibility in downstream analyses.

Given that OmnibusX is a self-funded commercial product, this represents the highest level of transparency possible while safeguarding intellectual property.

We have added a clarifying paragraph to the Design and Implementation section (Page 8, Lines 166–177), and a clarifying sentence to the Design and Implementation section (Page 5, Lines 98-101) to address this point.

- User Impact: If available, data on adoption, user feedback, or usability assessments could further demonstrate accessibility and impact.

We thank the reviewer for this suggestion and fully agree that adoption metrics and user feedback are valuable for demonstrating accessibility and impact. However, due to the nature of our user base, such information cannot be shared at this time. OmnibusX has only been established for one year, and publications from our academic users are still in progress. These users are typically engaged in ongoing analyses for manuscripts under preparation or peer review, and are therefore bound by institutional and journal policies that limit public disclosure prior to publication. Likewise, enterprise users are subject to strict internal policies that generally prohibit public recommendations or endorsements of specific software solutions.

- Future Plans: A brief roadmap for future developments and how user feedback informs updates would be a valuable addition.

We thank the reviewer for the suggestion. The Availability and Future Directions section now outlines our ongoing development priorities

Reviewer #3: OmnibusX aims to overcome challenges posed by fragmented analytical tools and high computational barriers by integrating workflows for various technologies (including bulk RNA-seq, single-cell RNA-seq, single-cell ATAC-seq, and spatial transcriptomics) into a single, cohesive application. The application incorporates existing open-source tools (such as Scanpy, DESeq2, SciPy, and scikit-learn) into transparent, reproducible pipelines, providing users with control over analysis parameters. However, this article suffers from the following problems.

We thank the reviewer for their constructive and positive feedback. We appreciate the recognition of our efforts to integrate workflows for multiple omics technologies into a unified application, as well as the acknowledgment of our use of established open-source tools within transparent and reproducible pipelines. Providing users with control over analysis parameters is a key objective of our platform, and we are pleased that the reviewer highlighted this aspect.

1. The paper mentions a proprietary cell type prediction module but fails to provide detailed information about its algorithmic principles, training data sources, and performance evaluation. Simply stating that it is "described in detail in a separate publication" is insufficient. The authors should at least outline the basic principles of the prediction engine, the type of model used, feature selection methods, and accuracy assessment results across different tissues and species.

We thank the reviewer for the opportunity to clarify this point. OmnibusX is transparent about its analytical workflow by specifying the computational packages used—whether open-source or proprietary—the stage at which they are applied, and the parameters available for user adjustment.

The proprietary cell type prediction engine is based on a curated database of 166 cell type and subtype-specific marker gene sets, compiled from 280 publications and validated across multiple datasets and tissues. The prediction algorithm builds on the AUCell method with additional steps to remove low-level background signals, calculate enrichment scores, and assign cell labels, followed by a smoothing step for unassigned cells based on nearest-neighbor consensus. While the curated marker sets and source code are proprietary to protect intellectual property and maintain competitive advantage, the methodology, algorithmic steps, and benchmarking results are published in detail in Truong et al., 2024. Users can manually adjust predictions or apply their own custom marker sets, ensuring flexibility and reproducibility in downstream analyses.

Given that OmnibusX is a self-funded commercial product, this represents the highest level of transparency possible while safeguarding intellectual property.

We have added a clarifying paragraph to the Design and Implementation section (Page 8, Lines 166–177) to address this point.

2. Although the paper mentions support for spatial transcriptomics data analysis, the description of relevant methods is relatively simplistic and lacks in-depth discussion of spatial-specific analytical techniques. It is recommended that the authors expand their description of spatial statistical methods.

We thank the reviewer for the suggestion to expand our discussion of spatial transcriptomics–specific analytical techniques. The current version of OmnibusX includes high-resolution image integration with gene expression overlays, interactive region annotation, and a ligand–receptor inference module leveraging curated interaction databases, as described in the manuscript.

We recognize that additional spatial statistical methods—such as spatially variable gene detection, neighborhood enrichment analysis, and spatial autocorrelation metrics—would further enrich OmnibusX’s capabilities. These features are part of our planned development roadmap and have now been explicitly mentioned in the revised Availability and Future Works section.

3. Despite the paper's title emphasizing "multi-omics," the methods section lacks specific technical descriptions of multimodal data integration analysis. It is recommended that the authors provide detailed explanations of how OmnibusX implements joint analysis of scRNA-seq with scATAC-seq, proteomics, or spatial transcriptomics data, particularly the implementation details of core technologies such as cross-modal feature alignment, multi-view clustering, and multi-omics factor analysis.

We appreciate the reviewer’s comments regarding the use of the term multi-omics in the manuscript title and the need to clarify how this relates to the current multimodal integration features. In the context of OmnibusX, multi-omics refers to the ability to analyze and visualize multiple omics data types—such as scRNA-seq, scATAC-seq, spatial transcriptomics, bulk RNA-seq, and TCR/BCR repertoire data—within a single, unified platform. This includes workflows for processing and jointly exploring paired multi-omic datasets generated from the same sample, such as scRNA-seq + TCR/BCR data (10x Genomics V(D)J + GEX), scRNA-seq + scATAC-seq (10x Multiome), and scRNA-seq + ADT (CITE-seq).

The current release supports shared visualization of features from multiple modalities on common low-dimensional embeddings (UMAP/t-SNE), clustering, marker identification, and annotation based on gene/protein accessibility or expression thresholds, metadata filters, or interactive selection tools. These capabilities allow users to explore concordance and divergence between modalities at the single-cell level.

We acknowledge that advanced multimodal statistical integration methods—such as cross-modal feature alignment, multi-view clustering, and multi-omics factor analysis—are not yet implemented. These are planned for future development and have been explicitly incorporated into the revised Availability and Future Works section.

4. The paper acknowledges that complex analyses still rely on external bioinformatics experts, but fails to provide clear solutions for situations with limited local computational resources. It is recommended that the authors discuss how to maintain data privacy while clearly specifying recommended hardware configurations and performance expectations for various types of analyses, helping users assess whether their equipment is suitable for specific analytical tasks.

We thank the reviewer for raising this important point. As described in the revised manuscript, OmnibusX is designed to preserve strict data privacy by performing all processing locally on the user’s hardware, with no transfer of input data or analytical results outside the user-controlled environment. Communication with the central OmnibusX server is strictly limited to user authentication, license validation, and downloading necessary reference files.

To address situations where users may have limited local computational resources, OmnibusX offers an enterprise edition that can be deployed on a private centralized server fully under the user’s control. This server can be hosted either on-premises within the institution’s infrastructure or on the user’s dedicated cloud environment (e.g., AWS, Google Cloud, or equivalent). In all cases, computations are executed entirely on hardware owned or managed by the user, ensuring that data remain within the institution’s administrative domain while enabling high-capacity computation.

For performance expectations, OmnibusX integrates core analytical functions from widely used open-source frameworks such as Scanpy and Seurat. Benchmarking results for Scanpy’s underlying methods are available in its original publication (Wolf et al., 2018), which provides detailed reference points for computational efficiency and scalability across different dataset sizes. This allows users to estimate hardware requirements for their analyses based on established and well-documented benchmarks.

5. To further enrich the content of the paper and demonstrate awareness of research frontiers, it is suggested that the authors include more references to graph representation learning and the integration of bioinformatics methods with large models. For example: "Interpretable identification of cancer genes across biological networks via transformer-powered graph representation learning" and "LLM-DDI: Leveraging Large Language Models for Drug-Drug Interaction Prediction on Biomedical Knowledge Graph."

We appreciate the reviewer’s suggestion and agree that graph representation learning and the integration of large models into bioinformatics pipelines represent important and rapidly developing areas of research. We have reviewed the suggested works and recognize their relevance in domains such as network-based gene prioritization and drug–drug interaction prediction.

However, these methodologies are not directly applicable to the current scope of OmnibusX or the specific focus of this manuscript, which centers on practical, privacy-centric, multi-omics analysis workflows for desktop and enterprise environments. Our current and near-term development priorities—outlined in the revised “Availability and Future Works” section—focus on expanding multi-omics data processing capabilities, enhancing interoperability, and refining spatial and multimodal analysis features.
